# Supplementary material for: Selecting optimal support grids for super-resolution cryogenic correlated light and electron microscopy
Source: Sci Rep. 2023 May 22;13:8270. doi: 10.1038/s41598-023-35590-x (PMC10203124; doi:10.1038/s41598-023-35590-x)
Supplement: Supplementary file 1 — Supplementary Information. [file 41598_2023_35590_MOESM1_ESM.pdf]

Supplementary information to:

Selecting optimal support grids for super-resolution cryogenic correlated light and electron microscopy

Mart G.F. Last, Maarten W. Tuijtel, Lenard M. Voortman, Thomas H. Sharp

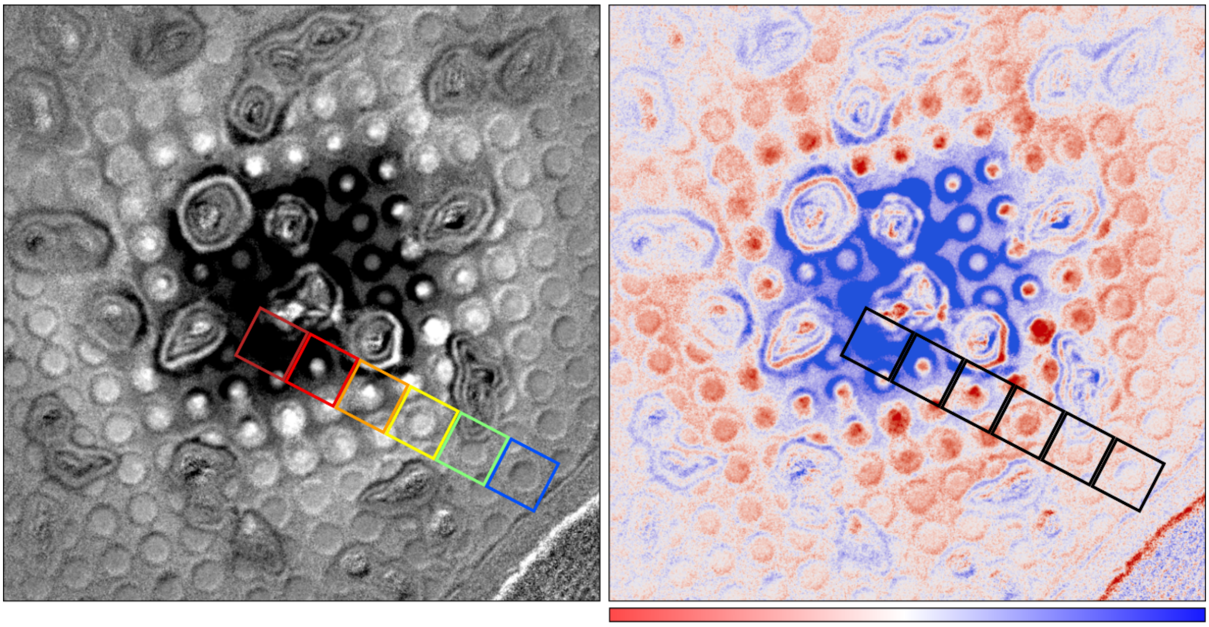

Supplementary Figure S1: difference maps showing the change in sample reflectivity due to illumination. These two images show the difference between the after-illumination and the pre-illumination reflected light images shown in main Figure 1. The data is the same in both images, but different look-up tables were applied. On the right, a diverging LUT was applied, so that the colour red corresponds to an increase in the reflectivity and blue to a decrease. Changes in the reflectivity correspond to changes in the structure of the sample: a decrease, as can be seen in the red squares, indicates that the ice was thinned or removed. An increase corresponds to the accumulation of crystalline ice particles, as can also be seen in main Figure 1. The change in the reflectivity is the smallest for the hole in the blue square, which, by virtue of close proximity to the grid bar, is most protected against reaching high temperatures.

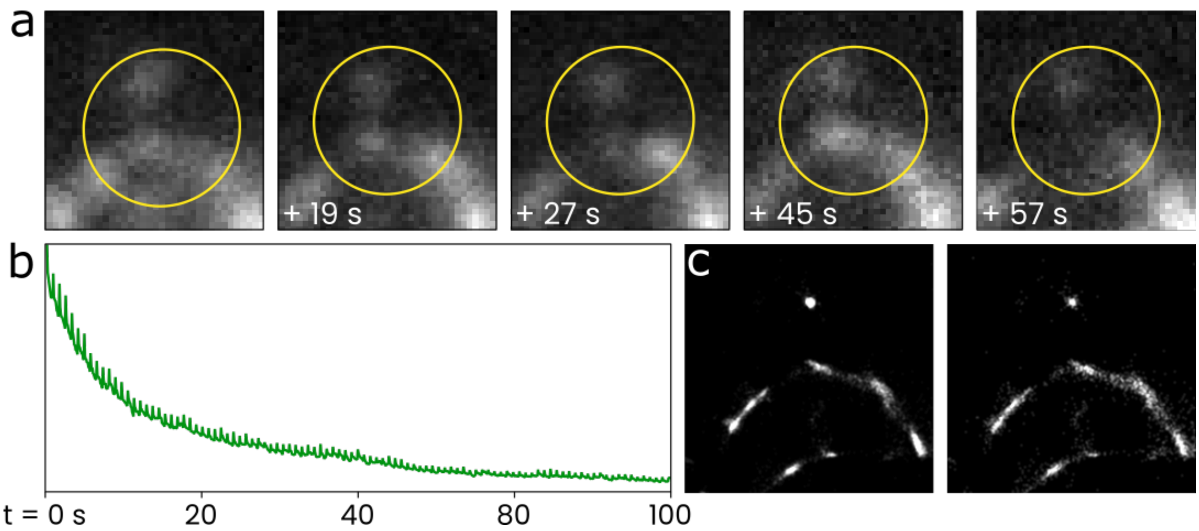

Supplementary Figure S2: a view of the raw data corresponding to the super-resolution reconstruction shown in main Figure 4. a) Single frames of the single-molecule localization microscopy timelapse video on which the super-resolution image in Figure 4 b/c-iii was based. **b)** Bulk fluorescence intensity vs. time. 405 nm illumination pulses were used to photo-activate rsEGFP2. These pulses can be recognized in the intensity trace: the sudden increases in fluorescence are caused by such a pulse. **c)** Two renderings of the same localization dataset: without particle merging (left), and with particle merging (right), where the maximum number of off-frames was 10 and the maximum linking distance 50 nm. Since the merging does not help (nor hinder) interpretation of the final image, we typically do not employ it.
